# Factors Influencing Job Satisfaction among Mental Health Nurses: A Systematic Review

**DOI:** 10.3390/healthcare12202040

**Published:** 2024-10-15

**Authors:** Ali Hudays, Faye Gary, Joachim G. Voss, Amal Arishi, Zainab A. Alfar, Ali M. Algodimi, Joyce J. Fitzpatrick

**Affiliations:** 1 Frances Payne Bolton School of Nursing, Case Western Reserve University, Cleveland, OH 44106, USA; fxg21@case.edu (F.G.); aaa428@case.edu (A.A.); jjf4@case.edu (J.J.F.); 2 Community, Psychiatric, and Mental Health Nursing Department, College of Nursing, King Saud University, Riyadh 13362, Saudi Arabia; 3 College of Nursing, University of Nebraska Medical Center, Omaha, NE 68198, USA; jovoss@unmc.edu; 4 Medical Surgical Department, College of Nursing, King Saud University, Riyadh 13362, Saudi Arabia; zalfar@ksu.edu.sa; 5 Nursing Department, Armed Forces Hospital Southern Region, Ministry of Defense, Khamis Mushait 62419, Saudi Arabia; aalgodimi@mod.gov.sa

**Keywords:** Herzberg’s two-factor theory, job satisfaction, mental health nurses, nurse satisfaction, psychiatric care settings

## Abstract

Background/Objectives: Job satisfaction is crucial for healthcare professionals, and understanding its influencing factors is essential for fostering a positive work environment, reducing turnover rates, and improving the overall quality of patient care. This systematic review examined the factors linked to nurse job satisfaction in psychiatric hospitals and diverse psychiatric settings, analyzing the findings through the lens of Herzberg’s theory. Despite existing evidence, gaps remain in understanding the differences in factors affecting job satisfaction across various settings. Methods: Following the Preferred Reporting Items for Systematic Reviews and Meta-Analysis guidelines, we conducted a comprehensive systematic review by searching six databases, namely PubMed, CINAHL, Cochrane Library, PsycINFO, Scopus, and APA PsycNet. Our search yielded an initial 567 studies published between January 2014 and February 2024, which were subsequently screened and evaluated based on predetermined inclusion criteria. Following this process, a total of 16 studies were deemed eligible for final analysis. Each of these selected studies underwent an independent review by two authors, utilizing the Joanna Briggs Institute checklist tool to ensure rigorous assessment. Results: The findings revealed that interpersonal relationships, working conditions, and recognition were the most frequently reported factors associated with nurse job satisfaction, along with various extrinsic, intrinsic, personal, emotional, and psychosocial factors. Notably, psychiatric hospitals showed a mix of personal, extrinsic, intrinsic, and psychosocial factors influencing job satisfaction, with demographic factors being the most frequently examined. In contrast, diverse psychiatric settings focused more on external aspects impacting job satisfaction. Conclusions: The review highlights the importance of both intrinsic and extrinsic factors and suggests that future research should employ more robust methods and consider psychiatric hospitals and other mental health contexts. Herzberg’s theory provides a valuable framework for understanding the factors associated with nurse job satisfaction.

## 1. Introduction

Job satisfaction is a crucial aspect of a healthcare professional’s career, and understanding the factors that influence it is essential to promote a positive work environment, reduce turnover rates, and enhance the overall quality of patient care. Job satisfaction can be defined as the emotional responses that an individual worker experiences in relation to factors such as their job, work experience, and the overall working environment [1]. Job satisfaction is essential for nurses to deliver high-quality care and maintain patient safety [2,3], and it can also boost nurse retention [4] and decrease turnover [5], thereby promoting a more stable and effective healthcare workforce. On the other hand, job dissatisfaction can have negative effects on the quality of care, patient safety, adverse events, patient dissatisfaction, and family complaints [6,7], ultimately compromising the well-being of both nurses and patients. Therefore, fostering job satisfaction is crucial for both professional success and optimal patient care.

A significant factor influencing job satisfaction among nurses is workplace violence. Research has demonstrated that nursing is the sector most significantly impacted by violence, placing nurses at the highest risk of aggression from patients, particularly in the form of threats to their physical safety and verbal abuse [8,9]. Furthermore, a considerable number of nurses have reported experiencing direct physical aggression [8,9]. Staempfli and Lamarche [10] argue that ensuring job satisfaction among nurses is essential not only for their well-being but also for enhancing patient safety and care quality, which in turn can lead to greater efficiency in healthcare [10]. The authors draw on Maslow’s hierarchy of needs and Herzberg’s two-factor theory to identify several key factors necessary for job satisfaction in this field. Notably, after salary, the need for job security and the absence of violence are identified as the most critical elements for achieving satisfaction [10].

The global nurse shortage, particularly in psychiatric nursing, is a pressing issue that may worsen job satisfaction concerns. Even before the COVID-19 pandemic, the worldwide nurse shortage was estimated to be around 5.9 million [11]. The International Council of Nurses (ICN) [12] predicts that an additional 13 million nurses will be needed globally by 2030 to address this shortage. In psychiatric nursing, the International Council of Nurses [13] estimates there are approximately 300,000 mental health nurses worldwide. However, the WHO Mental Health Atlas [14] reveals that only 31% of countries collect and analyze mental health data, making it challenging to understand the mental health nursing workforce and develop effective workforce planning strategies. In the United States, recent data from the American Psychiatric Nurses Association (APNA) indicates that there are currently around 35,000 practicing psychiatric–mental health nurse practitioners (PMHNPs), which increases to 39,374 when including psychiatric–mental health clinical nurse specialists (PMHCNSs) [15].

In the nursing field, various theories have been extensively examined to understand the key factors that contribute to job satisfaction among nurses. These theories include Hackman and Oldham’s job characteristics model, Herzberg’s two-factor theory, Maslow’s hierarchy of needs, and Vroom’s theory [16,17,18,19]. These frameworks provide valuable insights into the determinants of job satisfaction, enabling a deeper understanding of nurses’ motivations and needs.

Given its widespread recognition and extensive application in evaluating nursing job satisfaction in several studies [20,21,22,23,24], Herzberg’s two-factor theory was selected as the foundation for this review because of its unique contribution to understanding nursing job satisfaction. It is recognized as one of the most significant and popular theories in the study of job satisfaction [25]. While other models may have been used in several studies, Herzberg’s theory offers a distinct perspective by categorizing motivation factors into hygiene and motivator factors [26]. This categorizing provides a nuanced understanding of the factors that influence job satisfaction. Additionally, this theory has been widely applied and validated in various healthcare settings [27,28,29], making it a robust and relevant framework for examining nursing job satisfaction. By building on this theory, this review aims to provide a more comprehensive understanding of the factors that drive nursing job satisfaction among psychiatric nurses working in psychiatric hospitals and other mental health settings.

Herzberg and his colleagues introduced the two-factor model of work motivation in 1959 and further developed the motivation-hygiene theory, drawing inspiration from Maslow’s hierarchy of needs [30]. The framework comprises of two primary components, namely motivation and hygiene [26]. Motivation factors pertain to the elements that enhance job satisfaction, such as intrinsic factors like advancement opportunities, work itself, growth potential, responsibility, recognition, and achievement [26]. The hygiene factor encompasses various aspects that can contribute to job dissatisfaction. These factors, also known as extrinsic factors, include interpersonal relationships, salary, company policies and administration, relationships with supervisors, and working conditions [26]. According to the framework, the absence of hygiene factors was the main cause of job dissatisfaction, while the presence of motivators was the key contributor to job satisfaction.

Over the past decade, numerous reviews have explored factors influencing nurses’ job satisfaction across various countries and settings [31,32,33,34,35,36,37,38,39,40,41]. Despite this, no systematic review has specifically examined the factors affecting job satisfaction among nurses working in psychiatric hospitals. The absence of comprehensive exploration in this area is particularly notable, considering challenges and demands associated with psychiatric hospital work, which necessitate specialized skills, expertise, and support. This review aims to bridge this gap by comparing job satisfaction among psychiatric nurses in psychiatric hospitals and those in other mental health settings, such as outpatient clinics and psychiatric units in general hospitals. The work context significantly impacts nurses’ job satisfaction due to varying factors and expectations.

Our research aims to identify the key factors influencing job satisfaction among psychiatric nurses in psychiatric hospitals and other mental health contexts globally, and to compare these factors across different settings. Guided by the question “What factors contribute to job satisfaction among psychiatric nurses in psychiatric hospitals and other mental health contexts globally?”, we seek to provide a comprehensive understanding of the factors that drive job satisfaction among psychiatric nurses, and to explore how these factors may vary or align across different settings. This systematic review applies Herzberg’s theory of job satisfaction to interpret findings, and therefore, the study findings might inform healthcare policymakers and nurse leaders of targeted strategies to enhance job satisfaction and ultimately improve the quality of care in mental health settings.

## 2. Materials and Methods

### 2.1. Search Strategy

A comprehensive and systematic literature search was conducted, encompassing multiple electronic databases, including PubMed, CINAHL, Cochrane Library, PsycINFO, Scopus, APA PsycNet, and Google Scholar. The search strategy employed a carefully crafted set of keywords, including “mental health nurses”, “psychiatric nurses”, “Herzberg’s two-factor theory”, “motivation–hygiene theory”, “job satisfaction”, “nurse satisfaction”, “workplace satisfaction”, and “psychiatric care settings”, in conjunction with Boolean operators (“AND” and “OR”) to refine the search results. This targeted approach yielded a robust corpus of relevant journals aligned with the research question. To ensure the retrieval of contemporary and scholarly evidence, filters were applied to limit the search to peer-reviewed studies published between January 2014 and February 2024 to obtain up-to-date evidence. Limiting the search to studies published in the last 10 years can help to identify the most recent and relevant factors that contribute to job satisfaction among psychiatric nurses. Journal articles were selected based on predefined eligibility criteria (outlined in Table 1), thereby guaranteeing a rigorous and scholarly evidence base.

### 2.2. Inclusion and Exclusion Criteria

This systematic review adheres to a set of predefined inclusion and exclusion criteria to ensure the selection of relevant and high-quality studies. Studies were included if they were of a quantitative design, focused exclusively on mental health nurses as the population of interest, and conducted in psychiatric settings. Additionally, studies had to be written in English and published within the last 10 years (2014–2024). On the other hand, studies were excluded if they were systematic reviews, books, opinion articles, or of a non-quantitative design. Furthermore, studies involving healthcare professionals other than mental health nurses, mixed populations, or unrelated settings were also excluded. A detailed summary of the inclusion and exclusion criteria can be found in (Table 1).

### 2.3. Search Outcomes

**Figure 1:**
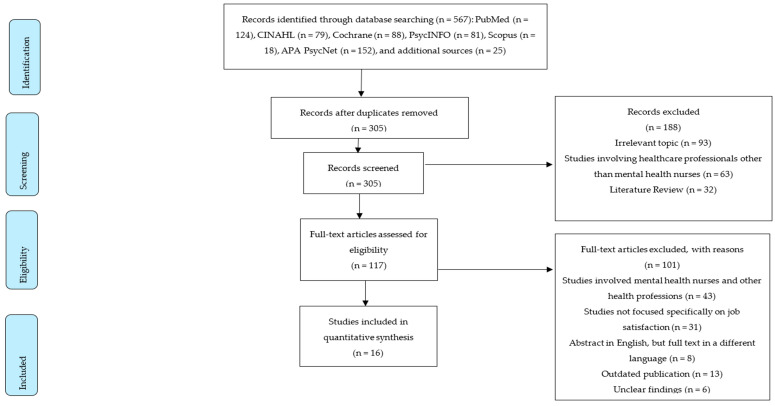
Prisma flow diagram.

Two reviewers (A.H. and A.A.) initially screened titles and abstracts to identify relevant citations that met the selection criteria. Two additional reviewers (F.G. and A.H.) conducted a comprehensive search and provided a detailed report of citations that fit the predefined criteria. Data extraction was performed by two evaluators, and verifications were conducted by two evaluators. Any discrepancies in the screening process were resolved through discussion, with a third reviewer’s input if necessary. Our search spanned six databases (PubMed, CINAHL, Cochrane Library, PsycINFO, Scopus, APA PsycNet) and Google Scholar, yielding 567 articles that underwent the selection process outlined in the PRISMA flow diagram [42]. Figure 1 illustrates the screening process. All articles were uploaded to the EndNote X9 software (version 9.3.3), Clarivate Analytics, Philadelphia, PA, USA. Available at: https://proquest.libguides.com/endnote (accessed on 26 May 2024). After uploading articles to EndNote X9 and removing duplicates, we screened titles and abstracts, excluding non-relevant articles. This left 117 full articles, of which 101 did not meet the inclusion criteria, resulting in a final sample of 16 articles included in this review, as shown in Figure 1 and Table 2.

### 2.4. Data Abstraction

Following the selection of 16 studies that met the inclusion criteria, a systematic data extraction process was conducted using a standardized table with predefined categories. These categories included: author and publication information, country, data collection period of the study, research design and framework, sampling strategy and size, setting, measures used (including internal and external validity considerations), statistical tests employed, key findings, and categorization according to Herzberg’s two-factor theory (Table 2). This structured approach ensured a comprehensive and transparent data extraction process.

### 2.5. Data Synthesis

This review utilized a narrative synthesis approach, combining textual and tabular methods to effectively summarize and evaluate the included studies. This approach allows for a thorough exploration of the findings, facilitating a systematic and transparent examination of the evidence. The subsequent section presents the findings in a clear and organized manner, laying the foundation for the discussion section. In the discussion section, the results are further analyzed to uncover connections, contradictions, and complexities. This nuanced understanding enhances comprehension of the research question, revealing the intricacies and subtleties of the topic. By integrating and interpreting the results, this review offers a comprehensive and contextualized understanding of the factors influencing job satisfaction among psychiatric nurses.

### 2.6. Quality Appraisal

The quality of the included studies [22,43,44,45,47,48,49,50,51,53,54,55,58,59,60,61] was assessed using the Joanna Briggs Institute (JBI) checklist tool [62] (Table 3). This tool, developed by the University of Adelaide’s Joanna Briggs Institute, is a validated and reliable instrument specifically designed for systematic reviews. Two independent evaluators (A.H. and F.G.) conducted the quality assessments, resolving any discrepancies through consensus and consultation with a third evaluator. According to the JBI tool, studies must meet all checklist criteria. Of the 16 included cross-sectional studies, 13 failed to account for all confounding factors, except two studies [48,51] which acknowledged these factors. One study lacked clear information [59]. Nine out of sixteen studies provided clear and explicit inclusion criteria. However, a notable exception was observed in six studies [22,44,47,50,60,61], which failed to explicitly define their exclusion criteria, potentially compromising the clarity and transparency of their methodology (Table 2 and Table 3). Furthermore, one study lacked essential information, omitting both inclusion and exclusion criteria as well as details about the sample characteristics [45]. Moreover, the exposure and outcome measures lacked clear demonstrations of reliability and validity [50,54], with one study clearly demonstrating reliability and validity for the exposure measure but not the outcome measure [51] (Table 3). Two studies did not employ appropriate statistical tests, which undermine the reliability of their findings [60,61]. Upon evaluation using the checklist, most studies included in this review were assessed to be of good quality except four studies, as summarized in Table 3.

## 3. Results

### 3.1. Characteristics of the Included Studies

This review encompasses 16 studies, collectively involving 11,828 participants, which were selected based on predetermined criteria. All studies employed cross-sectional designs, showcasing geographical diversity with investigations spanning 11 countries: the USA (n = 2), China (n = 2), Egypt (n = 4), Jordan (n = 1), Nigeria (n = 1), Israel (n = 1), Sweden (n = 1), Taiwan (n = 1), Japan (n = 1), South Africa (n = 1), and Singapore (n = 1). While a minority of studies (n = 3) were grounded in explicit theoretical or conceptual frameworks to assess job satisfaction, one of these studies was guided by both Quinn’s [56] Model of Managerial Leadership Roles and Mobley’s [57] Model of Employee Turnover to investigate how leadership styles impact job satisfaction and influence employees’ decisions to stay or leave their jobs. Most studies (n = 11) were conducted in psychiatric hospitals, followed by mixed mental health settings (n = 5). A comprehensive overview of the primary characteristics of the 16 reviewed studies is presented in Table 2, providing further insight into the study designs, populations, and settings.

### 3.2. Synthesize the Outcomes

#### 3.2.1. Comparison between Studies Conducted in Psychiatric Hospitals and Other Psychiatric Settings

This review encompasses a diverse range of study settings, with all included studies reporting data from either psychiatric hospitals, other psychiatric settings, or a combination of both. Notably, two studies employed a mixed design, collecting data from both psychiatric hospitals and other mental health settings, such as outpatient clinics, and departments in other different hospitals. Meanwhile, 11 studies focused exclusively on psychiatric hospital settings, and three studies were conducted solely in other mental health settings. This variation in study settings provides a rich foundation for exploring potential differences and similarities in job satisfaction among nurses working in distinct psychiatric care environments. The studies conducted exclusively in psychiatric hospitals revealed a mix of personal, extrinsic, intrinsic, and psychosocial factors influencing nurses’ job satisfaction. Notably, demographic factors were the most frequently examined factors in these studies, followed by extrinsic factors related to working conditions. While intrinsic factors were also mentioned, the focus was largely on the external aspects that impact nurses’ job satisfaction in diverse psychiatric settings. In contrast, studies conducted in other mental health settings exhibited a more balanced exploration of both intrinsic and extrinsic factors influencing nurses’ job satisfaction. While extrinsic factors were still prominent, with interpersonal relationships emerging as the most frequently examined aspect, intrinsic factors also received significant attention, led by the concept of responsibility as the most investigated intrinsic factor. Research conducted across mixed mental health settings, including psychiatric hospitals and other facilities, has tended to focus primarily on external influences on nurses’ job satisfaction, followed by internal or emotional aspects.

#### 3.2.2. Extrinsic Factors Affecting Job Satisfaction

Out of the total of 16 studies analyzed in this review, five indicated an association between interpersonal relationships and nurses’ job satisfaction, making the extrinsic factors most associated with job satisfaction. Findings of four studies showed that relations with peers, teamwork, and leadership roles led to a supportive and collaborative work environment and were therefore associated with job satisfaction [22,51,55,59]. A study found that the dimension of job satisfaction related to interpersonal relationships at work was significantly negatively correlated with job burnout, suggesting that positive workplace relationships are a crucial factor in reducing the risk of burnout and improving overall job satisfaction [48].

This review revealed that suboptimal work conditions, as reported by four studies, were a prominent contributor to nurses’ job dissatisfaction, ranking as the second most common external factor. Three studies identified job stress, burnout, and excessive work hours as significant predictors of reduced job satisfaction [53,54,59]. Notably, one study’s findings suggested that the time of day a nurse works has little impact on their job satisfaction, as no significant difference was found in satisfaction levels between day shift and evening shift nurses [43], suggesting that shift timing may not be a crucial factor in this context.

Adequate salary is a crucial aspect of job satisfaction, as it directly impacts an individual’s livelihood and well-being. The review highlighted the importance of salary in influencing job satisfaction, as three studies across various settings and countries consistently showed a positive correlation between salary and job satisfaction [22,43,59]. Salary was also identified as the third most common external factor contributing to nurses’ job satisfaction.

#### 3.2.3. Intrinsic Factors Affecting Job Satisfaction

Recognition is a vital aspect of job satisfaction, and acknowledging employees’ efforts and contributions can significantly boost their satisfaction levels. Three studies consistently demonstrated a positive correlation between perceived recognition at work and nurses’ job satisfaction [22,50,59], highlighting the significance of recognition in promoting job satisfaction among nurses.

According to two studies in this review, nurses’ job satisfaction was found to be positively linked to their level of responsibility [22,51]. Additionally, nurses who experienced opportunities for growth and advancement reported higher levels of job satisfaction [50,55]. This finding aligns with the notion that engaging in challenging work that demands skill development and utilization leads to increased job satisfaction [63,64]. As nurses take on more complex tasks and develop their professional skills, they tend to feel more fulfilled and satisfied with their job.

We found only one study that indicated a negative correlation between job satisfaction, specifically status at work, and job burnout [48].

#### 3.2.4. Personal and Emotional Factors Affecting Job Satisfaction

This review found that certain individual characteristics and personal attributes influence nurses’ job satisfaction, beyond aspects related to their job duties (intrinsic factors) or work environment (extrinsic factors). These personal factors, which encompass various aspects of a nurse’s personal and emotional makeup, have a significant impact on their job satisfaction. This review identified six studies that explored the link between demographic factors and job satisfaction among nurses, yielding mixed findings. In two studies, researchers found a positive relationship between age and job satisfaction. Specifically, Emmanuel Olatunde and Odusanya [44] revealed that respondents aged 40–59 years reported significantly higher job satisfaction, while Alsaraireh et al. [43] reported a positive correlation between age and job satisfaction, indicating that job satisfaction tends to increase with age, although a specific age range was not specified. In contrast, Zhou et al. [59] found a negative association between age and job satisfaction, suggesting that job satisfaction decreases with age. Payne et al. [54] did not find a significant difference in job satisfaction among different ranks, including professional nurses, registered nurses, and nursing assistants. Alsaraireh et al. [43] found that education level was negatively associated with job satisfaction, with associate degree holders reporting higher satisfaction than bachelor’s degree holders. Alsaraireh et al. [43] also found that female nurses and married nurses reported higher job satisfaction, while Emmanuel Olatunde and Odusanya [44] and Payne et al. [54] found no significant differences in job satisfaction based on gender or marital status. Alsaraireh et al. [43] discovered a positive association between years of experience and job satisfaction, but Payne et al. [54] found no significant association. Kabeel and Mosa Eisa [49] found a significant and positive correlation between professional identities encompassing traits such as professional image, assertiveness, self-responsibility, and job satisfaction (*p* = 0.000), indicating that nurses with stronger professional identities tend to experience higher levels of job satisfaction. Finally, according to Baum and Kagan [45], job satisfaction was higher among full-time nurses than part-time nurses.

The reason behind this disparity may be attributed to the fact that full-time psychiatric nurses derive higher job satisfaction from their sense of autonomy and control over their work, as well as the opportunities for specialization and variety in their patient care. In contrast, part-time psychiatric nurses attribute their lower job satisfaction to the limited opportunities for professional development and advancement, coupled with unpredictable schedules and workloads that can lead to increased stress and burnout.

Emotional factors were explored in three studies within this review. Notably, one study uncovered a significant negative correlation between burnout and job satisfaction (*p* = 0.0001), revealing that heightened emotional exhaustion, depersonalization, and diminished accomplishment are strongly linked to reduced job satisfaction [47]. This finding highlights the profound impact of emotional factors on job satisfaction, underscoring the importance of addressing burnout and its components to foster a more fulfilling work environment. Another study found that the nurses’ job satisfaction levels were negatively correlated with the frequency of experiencing negative emotions, suggesting that nurses who experienced negative emotions more frequently reported lower job satisfaction [53]. This finding complements the previous study, further emphasizing the significant impact of emotional factors on job satisfaction among nurses. The third study found that resilience was significantly and positively correlated with job satisfaction (*p* = 0.003) [58], indicating that nurses who exhibited higher levels of resilience tended to report greater job satisfaction. Resilience can be described as a dynamic process of effectively adapting to stress and challenging circumstances, involving the interplay between personal factors, environmental factors, and available resources in achieving positive outcomes [65].

#### 3.2.5. Psychosocial Factors Affecting Job Satisfaction

Out of the total of 16 studies analyzed in this review, three indicated an association between workplace violence and nurses’ job satisfaction. Zhou et al. [59] found a negative correlation between workplace violence and job satisfaction. They also reported that experiencing violence initiated by patients, whether through verbal abuse or physical assault, was negatively correlated with job satisfaction (*p* < 0.05).

In contrast, Mohamed Ali & Sayed Mohamed [61] found a statistically significant positive correlation among the total scores of workplace violence, perceived stress, and job satisfaction among psychiatric nurses (*p* < 0.001). This suggests that as exposure to workplace violence increases, both perceived stress and job satisfaction also tend to rise in this population.

AbdElhamid et al. [60] identified a strong statistically significant relationship between violence and job satisfaction, reporting correlation coefficients of R = 0.059 and R = 0.234, respectively, with a *p*-value of 0.000, indicating a positive association between the two variables. However, the study did not clarify the specific aspects of violence that these coefficients represent, which limits the interpretability of the findings and the understanding of their implications for job satisfaction.

Notably, this study revealed various correlations between job satisfaction, specific forms of aggression and recurrence of violence. Firstly, verbal aggression showed a weak positive correlation with job satisfaction (R = 0.105) and, similarly, a weak positive correlation with recurrence of violence (R = 0.081). In addition, physical aggression toward things presented a weak positive correlation with job satisfaction (R = 0.145), while also demonstrating a moderate positive correlation with recurrence of violence (R = 0.167) [60].

Furthermore, physical aggression toward self exhibited a moderate positive correlation with job satisfaction (R = 0.168) and a moderate positive correlation with recurrence of violence (R = 0.199). Lastly, physical aggression toward others indicated a moderate positive correlation with job satisfaction (R = 0.166) as well as a stronger moderate positive correlation with recurrence of violence (R = 0.206). Overall, all correlations were statistically significant at *p* = 0.005, highlighting the intricate relationships between job satisfaction, aggression, and the recurrence of violence in the workplace [60].

These findings from the two studies [60,61] might be attributed to several factors. First, the different measurement tools used for assessing job satisfaction and stress may influence the comparability of the results and the interpretation of the observed relationships. Additionally, the unique environment of psychiatric hospitals could contribute to the complex dynamics between workplace violence, perceived stress, and job satisfaction, suggesting that specific contextual elements play a significant role. Furthermore, nurses may develop coping strategies that allow them to maintain job satisfaction despite exposure to violence, indicating resilience within the workforce. Lastly, the intricate relationships between aggression and job satisfaction may reflect broader workplace dynamics, including team support and management practices, which warrant further exploration.

While we attribute the findings to several contextual factors, such as measurement tools and coping strategies, it is important to note discrepancies in the study conducted by AbdElhamid et al. [60]. Specifically, there are inconsistencies between the findings reported in the text and those presented in the results table. These discrepancies may hinder the clarity and reliability of the study’s conclusions, raising concerns about the validity of the reported data. Such inconsistencies make it challenging for readers to accurately interpret the findings and may undermine confidence in the study’s overall contributions.

## 4. Discussion

Nurses’ job satisfaction is a critical indicator of healthcare workforce outcomes, influencing patient care, nurse retention, and organizational performance. This systematic review aims to provide a comprehensive overview of the empirical evidence on the factors influencing nurses’ job satisfaction, synthesizing findings from studies published between January 2014 and February 2024. Our analysis, guided by Herzberg’s two-factor theory, categorizes these factors into extrinsic and intrinsic motivators. Notably, personal, emotional, and psychosocial factors, not explicitly addressed by Herzberg’s framework, also emerged as significant contributors to job satisfaction. While our focus is on psychiatric care nurses, specifically those working in psychiatric hospitals, we also explore job satisfaction factors across various mental healthcare environments. Employing statistical techniques like linear regression and Pearson correlation, most studies provide a quantitative lens on this issue. By synthesizing and standardizing data from multiple studies, our review reveals that psychiatric nurses generally hold a positive, albeit nuanced, view of their work, with their satisfaction levels falling within the middle range of the spectrum. The analysis revealed that the studies most commonly explored four categories of factors influencing job satisfaction: extrinsic factors, which encompass workplace dynamics (interpersonal relationships, working conditions) and compensation (salary); intrinsic factors, which include autonomy and sense of purpose (recognition and responsibility); personal factors, comprising demographic characteristics (age, gender, marital status, and educational level); and psychosocial factors, which include verbal abuse, and physical aggression. These studies were conducted in various mental health settings, including psychiatric hospitals, other psychiatric facilities, and a mix of both.

### 4.1. Comparison between Studies Conducted in Psychiatric Hospitals and Other Psychiatric Settings

The findings of this study indicate that, among nurses in psychiatric hospitals, extrinsic factors pertaining to working conditions are the most frequently examined, with aspects such as work shift, job stress, burnout levels, and work hours, as well as salary, which is also a prominent area of investigation. This is followed by personal factors, including age, which are also explored in relation to job satisfaction. Notably, intrinsic factors, specifically recognition, emerge as a significant consideration, encompassing facets such as social recognition, feedback prior to disciplinary action, and acknowledgment of employees’ additional credentials.

In contrast, our analysis of a mixed range of psychiatric settings revealed a different pattern of factors associated with job satisfaction. Specifically, while personal factors received limited attention, interpersonal relationships, including leadership dynamics, collegial connections, and effective communication, emerged as a primary focus. Moreover, intrinsic factors, particularly the sense of responsibility, played a significant role in predicting job satisfaction, encompassing feelings of being supported and cared for in the work environment, as well as being accountable and in control of one’s work. These findings suggest that fostering positive interpersonal relationships and a sense of responsibility may be key to enhancing job satisfaction in mixed psychiatric settings.

The disparity in findings between the two settings (psychiatric hospitals and mixed psychiatric settings) can be attributed to variations in setting characteristics, nurse roles and responsibilities, organizational culture, and nurse demographics and experiences. These differences likely influence the prioritization of extrinsic factors like working conditions in psychiatric hospitals versus the emphasis on interpersonal relationships and communication in mixed psychiatric settings. Understanding these contextual factors is crucial for tailoring strategies to enhance job satisfaction and nurse well-being.

### 4.2. Extrinsic and Intrinsic Factors Affecting Job Satisfaction

Our review is the first of its kind to explore the factors influencing nurses’ job satisfaction in psychiatric hospitals and other mental health contexts, and our results align with the findings of previous studies. For example, Ayalew et al. [66] found that nurse job satisfaction is associated with intrinsic factors such as achievement and recognition at work. Another study found that career growth is the most important intrinsic factor affecting job satisfaction, followed by responsibility at work [67], while also highlighting monthly compensation as the most significant extrinsic factor contributing to job satisfaction among nurses.

Moreover, according to a study conducted by Hellín Gil et al. [68], the World Health Organization (WHO), the International Council of Nurses (ICN), and the International Labour Organization (ILO) have identified that inadequate working conditions can have an impact on the job satisfaction of nurses. Previous studies investigating the influence of intrinsic and extrinsic factors on job satisfaction among nurses have predominantly focused on settings other than psychiatric settings, such as ICUs, health centers, or general medical wards. Consequently, there is a clear need for further research in this particular area to bridge the existing gap and gain a better understanding of the factors impacting job satisfaction among psychiatric nurses. The above-mentioned findings highlight the relationships between nurses’ job satisfaction and a range of external and internal factors. However, it is important to acknowledge that previous research conducted on psychiatric nurses, particularly in psychiatric hospitals and mixed psychiatric settings, has primarily concentrated on examining external factors at the expense of intrinsic one.

According to Herzberg’s seminal work [26], hygiene factors are extrinsic to the job itself and serve to prevent job dissatisfaction by addressing the need to avoid unpleasantness in the work environment and workplace [26]. These factors, if present, can mitigate discontent but do not necessarily foster positive attitudes towards the job. In contrast, motivation factors, or motivators, are intrinsic to the job and have the power to inspire positive attitudes and job satisfaction by fulfilling the need for growth and self-actualization [26]. By satisfying these deeper needs, motivators can lead to a sense of purpose and fulfillment, driving employees to excel in their roles. It follows that researchers in the field of nursing should explore the impacts of various strategies related to intrinsic factors specifically in psychiatric nursing settings. By addressing this existing research gap and focusing on promoting nurses’ job satisfaction, valuable insights can be gained.

### 4.3. Personal and Emotional Factors Affecting Job Satisfaction

Our review uncovered key demographic factors linked to mental health nurses’ job satisfaction, highlighting the need to target specific groups, including single individuals, younger nurses, males, and those with limited experience, who appear more prone to job dissatisfaction. However, the impact of gender, marital status, and educational level on job satisfaction remains unclear, underscoring the need for further investigation. Our findings resonate with a recent review of Saudi Arabian hospital nurses [37], which similarly found inconsistent results regarding gender and educational level. Maqbali [69] identified a range of factors that may impact job satisfaction, including an individual’s level of experience, educational attainment, work setting, age, and gender [69]. Similarly, Chien and Yick [70] found associations or impacts between nurses’ job satisfaction and individual predisposing characteristics, including age, years of experience, and education level [70]. These findings underscore the significance of personal and demographic variables, encompassing individual characteristics and professional experiences, in shaping nurses’ overall job satisfaction.

Our synthesis of the literature reveals that emotional factors, including burnout, negative emotions, and resilience, are significantly correlated with psychiatric nurses’ job satisfaction, with consistent correlations emerging across the studies we examined. Our results align with existing research, e.g., with Shahrbabaki et al. [71], who consistently showed a positive correlation between resilience and nurses’ job satisfaction. Specifically, the researchers found that higher resilience was associated with higher job satisfaction among nurses, and they also observed this relationship for specific dimensions of resilience, including trust in individual instincts, tolerance of negative affect, positive acceptance of change and secure relationships, and spiritual influences [71]. In contrast, Heidari et al. [72] revealed that burnout, specifically its dimensions of emotional exhaustion and personal accomplishment, exhibited a significant negative association with nurses’ job satisfaction, highlighting the importance of addressing these factors to promote a positive work environment.

These findings underscore the need for healthcare organizations to prioritize nurse well-being and implement evidence-based strategies to foster resilience and mitigate burnout. To boost job satisfaction, healthcare organizations should promote stress management techniques, a healthy work–life balance, and opportunities for professional growth. Additionally, fostering a supportive work environment and acknowledging contributions can significantly enhance resilience. Professional development programs focusing on teamwork [73], and multidisciplinary support, including the involvement of families and colleagues [74], can further strengthen nurses’ resilience, leading to improved job satisfaction and patient care. By addressing these emotional factors, healthcare organizations can promote a culture of resilience, benefiting both nurses and patients. Future research should continue to explore effective interventions and strategies to support nurses’ emotional well-being and promote optimal patient care.

### 4.4. Psychosocial Factors Affecting Job Satisfaction

Our synthesis of the literature reveals that psychosocial factors, including verbal abuse and physical aggression associated with workplace violence, are significantly correlated with psychiatric nurses’ job satisfaction. The findings illustrate a complex relationship between workplace violence and job satisfaction among nurses. While some studies indicate negative impacts, some suggest positive correlations, highlighting the need for further investigation into these nuanced relationships.

Specifically, our review found a negative correlation between workplace violence and job satisfaction. Experiencing violence initiated by patients, whether through verbal abuse or physical assault, was negatively correlated with job satisfaction. This finding aligns with earlier studies by Chang & Cho [75] and Cheung & Yip [76], who also reported that violence from patients adversely affects job satisfaction.

Notably, our review uncovered an unexpected finding: a significant positive correlation among workplace violence, perceived stress, and job satisfaction among psychiatric nurses. As scores for workplace violence and perceived stress increased, job satisfaction also tended to rise. This relationship challenges conventional expectations and suggests a more complex dynamic at play. As highlighted in the results, the unique environment of psychiatric hospitals may play a significant role in this complexity by promoting coping mechanisms that help nurses build resilience. As a result, nurses can find meaning in their work, even when faced with significant stressors. The statistical significance (*p* < 0.001) underscores the reliability of this association and prompts further exploration into the underlying mechanisms that may contribute to these surprising dynamics.

Understanding these complexities is crucial for healthcare organizations aiming to improve nurses’ job satisfaction and overall well-being. Future research should explore the reasons behind the positive correlation, as well as investigate effective interventions to mitigate workplace violence and its impacts on nurses. Additionally, it is crucial for institutions to establish comprehensive policies that specifically address workplace violence. This includes implementing patient rights documents and ensuring employee rights are upheld to create a safer working environment. Providing de-escalation training for staff and maintaining adequate staffing levels are also important strategies that can help prevent incidents of violence and reduce stress among nurses.

### 4.5. Strengths and Limitations

This study has several strengths, including its comprehensive review of the literature on factors influencing job satisfaction among psychiatric nurses, its use of a clear and established theoretical framework (Herzberg’s two-factor theory), and its inclusion of psychosocial factors (such as verbal abuse and physical aggression) and emotional factors (such as burnout, negative emotions, and resilience), which are often overlooked in studies on job satisfaction. Additionally, the study’s comparison across different psychiatric settings provides valuable insights into the generalizability of the results, and its practical recommendations for healthcare organizations offer a clear path forward for improving job satisfaction among psychiatric nurses. However, it is important to note that while this review identifies key factors influencing job satisfaction, it does not delve into specific strategies for addressing these factors, indicating a need for further exploration in future research. The inclusion of response rates in most studies strengthens the findings by providing a clear indicator of participant engagement and representation. The assessment of study quality using a standardized tool reveals that most studies demonstrate robust methodological rigor, enhancing the validity and reliability of the findings. Moreover, the diverse measurement tools employed to assess predictors and outcome variables, along with reported validity and reliability, ensure a comprehensive and transparent examination of the relationships between variables. Despite the study’s contributions, it is essential to acknowledge some limitations. One key limitation is the language restriction, as only English-language papers were considered, potentially overlooking relevant research published in other languages. A further significant limitation is the predominance of methodological weaknesses, as all included studies employed cross-sectional designs. While most of these studies utilized convenience sampling, this approach hinders the ability to establish causal connections and draw definitive conclusions.

### 4.6. Burnout, Stress, and Negative Emotions in Nurses: Implications for Healthcare

The review highlighted the significant impact of emotional factors, such as burnout, negative emotions, and lack of resilience, on job satisfaction among nurses. Specifically, the research found a strong negative correlation between burnout and job satisfaction, indicating that heightened emotional exhaustion, depersonalization, and diminished sense of personal accomplishment are strongly linked to reduced job satisfaction in nurses.

This is concerning, as burnout and negative emotions can have far-reaching consequences for healthcare quality and patient outcomes. Burned-out and stressed nurses are more prone to making errors, providing lower-quality care, and experiencing job dissatisfaction and turnover [6,77,78,79]. This can lead to long-term issues such as poorer patient safety, increased medical errors, and reduced continuity of care. Additionally, specific emotions such as depression and anxiety are linked to poorer overall health outcomes, as well as increased risk of developing specific medical conditions such as cancer and heart disease [80]. On the other hand, positive emotions have been associated with better general health.

To address this critical issue, healthcare organizations should implement evidence-based interventions to support nurses’ emotional well-being, strengthen safety measures, and build resilience. Potential strategies include promoting stress management and self-care, fostering a culture of resilience, enhancing work–life balance, and offering mental health support. Additionally, organizations should focus on enhancing interpersonal relationships by implementing team-building activities and communication workshops, improving working conditions through adequate staffing levels, and developing structured recognition programs to acknowledge nurses’ contributions. By addressing both the emotional factors and the broader aspects that impact nurses’ job satisfaction, healthcare organizations can promote a healthier, more resilient workforce and ultimately enhance the quality of patient care. Investing in the well-being of nurses is crucial for creating a sustainable and high-performing healthcare system.

## 5. Conclusions

Although existing research has explored the factors linked to nurses’ job satisfaction, our review has identified crucial factors that require further exploration. To thoroughly understand the influence of these factors, it is essential to conduct additional studies utilizing more rigorous methodologies. A notable gap remains in understanding how job satisfaction factors differ between psychiatric hospitals and other mental health settings. Research in this area could allow organizations to adapt their strategies for improving nurses’ job satisfaction according to specific work environments. Our findings emphasize the significance of interpersonal relationships and working conditions as crucial extrinsic factors, alongside recognition and responsibility at work as key intrinsic factors affecting job satisfaction among nurses. In examining psychosocial factors such as verbal abuse and physical aggression associated with workplace violence, we conclude that there is a knowledge gap regarding their impact on psychiatric nurses’ job satisfaction. Our synthesis of the literature reveals that these factors are significantly correlated with job satisfaction, illustrating a complex relationship. While some studies indicate negative impacts, others suggest positive correlations, highlighting the need for further investigation into these nuanced relationships. In response to these findings, nursing managers and hospital administrators should develop and implement strategies that enhance both intrinsic and extrinsic factors, and those related to psychosocial factors. It is crucial to recognize that relying solely on either type of factor will not adequately address job dissatisfaction among nurses. For instance, addressing psychosocial factors such as verbal abuse and physical aggression is essential for creating a supportive work environment. Hospital management should strive for a balanced integration of both intrinsic and extrinsic elements, along with psychosocial factors such as workplace violence, including verbal abuse and physical assault. This approach should be tailored to the unique needs of their nursing staff and aligned with the organization’s commitment to fostering a healthy work environment. Implementing interventions like conflict resolution training, peer support programs, and robust policies against workplace violence can significantly improve nurses’ job satisfaction. This review supports Herzberg’s theory, offering a valuable framework for understanding the dynamics of intrinsic and extrinsic factors in relation to job satisfaction, while also highlighting the critical role of psychosocial factors in shaping the nursing work experience. This review supports Herzberg’s theory, providing a valuable framework for understanding the interplay between intrinsic and extrinsic factors in relation to job satisfaction. Additionally, future studies should consider the critical role of psychosocial and emotional factors, such as workplace violence and emotional support, in shaping the nursing work experience. A comprehensive approach that incorporates these factors will enhance the understanding of job satisfaction among nurses.

## Figures and Tables

**Table 1:** Summary of literature search based on inclusion criteria.

Inclusion Criteria	Exclusion Criteria	Key Words	Databases Searched	Number of Studies	
Studies that are of a quantitative type.	Studies that are of a qualitative type, systematic reviews, books, and opinion articles.	(Mental Health Nurses OR Psychiatric Nurses OR Nurses in Psychiatric Settings OR Nurses in Mental Health Care) AND (Herzberg’s Two-Factor Theory OR Job Satisfaction OR Motivation–Hygiene Theory OR Two-Factor Theory) AND (Psychiatric Care Settings OR Mental Health Facilities OR Inpatient OR Outpatient OR Community Mental Health OR Acute Care OR Long-term Care) AND (Job Satisfaction OR Work Satisfaction OR Workplace Satisfaction OR Employee Satisfaction OR Nurse Satisfaction OR JS).	PubMed	124	
Studies exclusively focusing on mental health nurses as the population of interest in relation to job satisfaction.	Studies involving healthcare professionals other than mental health nurses (e.g., physicians, therapists, etc.). Additionally, studies involving both healthcare professionals and nurses (mixed populations) are excluded.		CINAHL	79	
Studies settings should be at psychiatric settings.	Studies that do not specifically investigate mental health care or psychiatric settings.		Cochrane Library	88	
Studies written in English.	Studies written in languages other than English.		PsycINFO	81	
Studies published in the last 10 years.	Studies published before 2014.				
			Google Scholar	25	
			Scopus	18	
			APA PsycNet	152	
				Total: 567	

**Table 2:** Characteristics and outcomes of the included studies.

Authors/Year/Data Collection Period/Country	Design	Framework	Sample Strategy/Size/Setting	Measures/Internal and External Validity	Type of Statistical Test	Findings Related to Job Satisfaction among Nurses	Factor Category
[43] N/S Jordan	Descriptive cross-sectional design	_	Strategy: non-probability sampling “convenience sampling”. Size: Of 200 nurses, a total of 154 nurses completed the questionnaires (inclusion and exclusion criteria were provided). Setting: Governmental hospital for mental health in Jordan.	The Minnesota Job Satisfaction Questionnaire (MSQ). Cronbach’s alpha = 0.91. Internal Validity Concern: Instrumentation: Not a problem. External Validity Concern: Response Rate: Not a problem; the study reported a response rate of 86%.	Independent-sample *t*-tests	Females reported much higher job satisfaction scores (mean = 64.07, SD = 8.88) than males (mean = 55.40, SD = 11.08), t (152) = −5.39, *p* = 0.000).	Personal
						Married people reported higher satisfaction scores (mean = 63.10, SD = 10.41) compared to single people (mean = 55.95, SD = 10.03), with t (152) = −4.263, *p* = 0.000).	Personal
						Participants with an associate degree reported higher satisfaction scores (mean = 61.45, SD = 10.60) compared to those with a bachelor’s degree (mean = 54.18, SD = 9.85), with t (152) = 3.324, *p* = 0.001).	Personal
						No significant difference between day shift and evening shift t (152) = 1.551, *p * = 0.123).	Work conditions (Hygiene)
					Pearson correlations	Strong and significant positive correlation between age and job satisfaction (r = 0.508, *p* = 0.01).	Personal
						Strong and significant positive correlation between experience and job satisfaction (r = 0.566, *p* = 0.01).	Personal
						Strong and significant positive correlation between salary and job satisfaction (r = 0.780, *p* = 0.01).	Salary (Hygiene)
[44] June 2013 Nigeria	Descriptive cross-sectional study	_	Strategy: Random sampling Size: Sample size was 110 mental health nurses (inclusion criteria provided, exclusion criteria unclear). Setting: Neuropsychiatric Hospital, Aro, Abeokuta, Nigeria	The Minnesota Satisfaction Questionnaire (MSQ). Internal Validity Concern: Instrumentation: Reliability & validity unreported, threatening internal validity. External Validity Concern: Response Rate: Not a problem; the study reported a response rate of 96.5%.	Chi-Squared test	Older respondents (aged 40–59 years), of whom 44.4% reported high job satisfaction levels, showed significantly higher levels of job satisfaction compared to younger respondents (aged 20–39 years), of whom 27.7% reported high job satisfaction (χ^2^ = 9.59, *p* = 0.024).	Personal
						No significant difference for marital status, number of children, work cadre, religion, and length of years in service.	Personal
[45] N/S Israel	Cross-sectional quantitative design study	_	Strategy: non-probability sampling “convenience sampling”. Size: 52 nurses (inclusion & exclusion criteria were not explicitly provided). Setting: A large psychiatric hospital in central Israel. Specifically, nurses working on: Closed wards Open wards.	Toren, Kerzman, and Kagan [46] instrument. Cronbach’s alpha = 0.81. Internal Validity Concern: Instrumentation: Not a problem. External Validity Concern: Response Rate: Not a problem; the study reported a response rate of 95%.	Independent-sample *t*-tests	Level of job satisfaction was notably greater among full-time nurses compared to those who worked part-time (t = 2.05, *p* < 0.05).	Personal
[47] N/S Egypt	Descriptive correlation design	_	Strategy: Not stated. Size: The sample for the study comprised 50 staff nurses (inclusion criteria provided, exclusion criteria unclear). Setting: Psychiatric Department in Tanta University Hospital and Tanta Mental Health Hospital, Egypt.	Kuopio University Hospital Job Satisfaction Scale (KUHJSS). Cronbach’s α = 0.76 Maslach Burnout Inventory Scale (MBI). Cronbach’s α = 0.76 Internal Validity Concern: Instrumentation: Not a problem External Validity Concern: Based on the characteristics table and findings, it appears that all 50 nurses returned the questionnaire. However, the authors did not explicitly state the response rate.	Pearson’s correlation coefficient	Significant negative correlation (r = −0.555, *p* = 0.0001) between total burnout score and job satisfaction score, specifically in terms of emotional exhaustion, depersonalization, and accomplishment.	Personal/Emotional factors
[22] N/S Sweden	Cross-sectional survey	Herzberg’s motivation–hygiene theory	Strategy: non-probability sampling “convenience sampling”. Size: Out of 130 nurses employed at the clinic, 118 completed the survey (inclusion criteria provided, exclusion criteria unclear). Setting: A psychiatric university hospital clinic located in western Sweden.	The researchers designed a custom-made survey comprising 69 questions. Cronbach’s alpha (0.71–0.89). Internal Validity Concern: Instrumentation: Not a problem. External Validity Concern: Response Rate: Not a problem; the study reported a response rate of 90.7%.	Multiple regression	Relationships with peers exhibited a significant correlation with job satisfaction (B = 0.263, SE = 0.071, Beta = 0.284, t = 3.704, *p* = 0.001).	Interpersonal relationship (Hygiene)
						Salary demonstrated a significant correlation with job satisfaction (B = 0.157, SE = 0.06, Beta = 0.120, t = 2.574, *p* = 0. 019).	Salary (Hygiene)
						Recognition displayed a positive correlation with job satisfaction (B = 0.298, SE = 0.108, Beta = 0.359, t = 2.759, *p* = 0.012).	Recognition (Motivation)
						Responsibility showed a positive correlation with job satisfaction (B = 0.109, SE = 0.058, Beta = 0.149, t = 1.879, *p* = 0.023).	Responsibility (Motivation)
[48] November to December 2021 China	Cross-sectional descriptive study	_	Strategy: Simple random sampling Size: Of the 229 questionnaires distributed, 212 were completed and returned (inclusion and exclusion criteria were provided). Setting: The study was conducted among psychiatric nurses working in psychiatric units in four Grade-A tertiary hospitals in Jiangsu Province, China.	Job satisfaction scale. Cronbach’s alpha ranged from 0.916, to 0.969 (two dimensions). The MBI-GS job burnout scale. Cronbach’s alpha values of 0.911 for the overall scale, and 0.956, 0.965, and 0.953 for the emotional exhaustion, depersonalization, and low personal accomplishment subscales, respectively. Internal Validity Concern: Instrumentation: Not a problem External Validity Concern: Response Rate: Not a problem; the study reported a response rate of 92.58%.	Pearson correlation	Significant negative correlation between satisfaction with “Status at Work” and job burnout (r = −0.415, *p* < 0.000).	Status (Motivation)
						Significant negative correlation between satisfaction with “Interpersonal Relationships at Work” and job burnout (r = −0.220, *p* = 0.019).	Interpersonal relationship (Hygiene)
[49] February 2016 to March 2016 Egypt	Descriptive correlation research design.	_	Strategy: non-probability sampling “convenience sampling”. Size: 50 nurses (inclusion and exclusion criteria were provided). Setting: Abbassya Mental Health Hospital in Egypt.	Mueller and McCloskey Satisfaction Scale. Cronbach’s alpha = 0.85. Nurses’ professional identity tool. Cronbach’s alpha = 0.96. Internal Validity Concern: Instrumentation: Not a problem. External Validity Concern: Response Rate: Based on the characteristics table and findings, it appears that all 50 nurses returned the questionnaire. However, the authors did not explicitly state the response rate.	Pearson correlation	Statistically significant positive correlations between total nurses’ professional identity scores and nurses’ satisfaction, with a correlation coefficient of r = 0.589. Additionally, the relationship between nurses’ professional identity and nurses’ satisfaction was statistically significant, with a *p*-value of 0.000.	Personal
[50] N/S Western US	Descriptive, cross-sectional study.	_	Strategy: non-probability sampling “convenience sampling”. Size: From a total of 260 nurses, only 94 RNs completed the online survey (inclusion criteria provided, exclusion criteria unclear). Setting: A large inpatient state-run psychiatric hospital in the western US.	Custom-made questionnaire Internal Validity Concern: Instrumentation: Reliability & validity unreported, threatening internal validity. External Validity Concern: Response Rate: The study reported a low response rate of 36%.	Pearson correlations	Employees who receive feedback before disciplinary action is taken tend to have higher job satisfaction (*p* < 0.05).	Recognition (Motivation)
						Recognizing and acknowledging employees’ additional credentials is linked to higher job satisfaction with (*p* < 0.05).	Recognition (Motivation)
						Providing employees with opportunities for growth and development is significantly related to higher job satisfaction with (*p* < 0.05).	Growth (Motivation)
[51] 2013 Taiwan	Correlational cross-sectional design.	_	Strategy: Stratified random sampling. Size: 185 nurses (inclusion and exclusion criteria were provided). Settings: Diverse range of acute care institutions across Taiwan, including academic medical centers (n = 4), metropolitan hospitals (n = 9), and district hospitals (n = 12), with a specific focus on acute psychiatric wards and psychiatric nurses providing care to patients with mental health conditions.	Job satisfaction was evaluated using a single item adapted from Yoder [52]. Internal Validity Concern: Instrumentation: Reliability & validity unreported, threatening internal validity. Indicators of a quality nursing work environment. Internal Validity Concern: Cronbach’s α = 0.93 Instrumentation: Not a problem External Validity Concern: Response Rate: Not a problem; the study reported a response rate of 99%.	Hierarchical regression models after adjusting for variables	Professional specialization and teamwork were positively related to job satisfaction (t = 2.496, β = 0.203, B = 0.170, *p* < 0.05). Support and caring in the work environment were positively related to job satisfaction (t = 5.531, β = 0.592, B = 0.514, *p* < 0.001).	Interpersonal relationship (Hygiene) Responsibility (Motivation)
[53] June to August 2017 Japan	Correlational cross-sectional design.	Conceptual framework	Strategy: Random sampling. Size: From a total of 1097 nurses, 663 psychiatric nurses responded (inclusion and exclusion criteria were provided). Setting: 13 psychiatric hospitals in the Chugoku area.	Negative Feeling Toward Patient Frequency scale. Cronbach’s α = 0.953. Support in workplace scale. Cronbach’s α: SW (supervisors) = 0.976; SW (co-workers) = 0.971. The new Brief Job Stress Questionnaire (job stress and satisfaction). Cronbach’s α: (job satisfaction) = 0.722; (job stress) = 0684). Internal Validity Concern: Instrumentation: Not a problem External Validity Concern: Response Rate: Not a problem; the study reported a response rate of 60.4% (663 out of 1097), and of those respondents, 87.0% (577 out of 663) provided valid responses without missing data.	Logistic regression	Nurses’ job satisfaction levels were positively correlated with support from supervisors (B = 0.067, SE = 0.009, *p* = 0.000, OR: 1.069).	Supervision (Hygiene)
						Nurses’ job satisfaction levels were negatively correlated with job stress levels (B = −0.287, SE = 0.038, *p* = 0.000, OR: 0.751).	Working conditions (Hygiene)
						Nurses’ job satisfaction levels were negatively correlated with the frequency of experiencing negative emotions (B = −0.020, SE = 0.005, *p* = 0.000, OR: 0.980).	Personal/Emotional factor
[54] Between November and December 2017 South Africa	Cross-sectional study	_	Strategy: Not stated Size: From a total of 221 psychiatric nurses, 132 psychiatric nurses participated (inclusion and exclusion criteria were provided). Setting: Stikland Psychiatric Hospital in Belville.	Job Satisfaction Survey (JSS). Copenhagen Burnout Inventory (CBI). Internal Validity Concern: Instrumentation: Reliability & validity unreported, threatening internal validity. External Validity Concern: Response Rate: Not a problem; the study reported a response rate of 60%.	Linear regression	Higher levels of burnout were significantly associated (r = −0.077, *p* < 0.01) with lower levels of job satisfaction (Negative correlation).	Working conditions (Hygiene)
						No significant associations between job satisfaction and gender.	Personal
						No significant associations between job satisfaction and rank.	Personal
						No significant associations between job satisfaction and years of experience.	Personal
[55] N/S USA	Correlational, exploratory design	Quinn’s [56] Model of Managerial Leadership Roles and the conceptual framework of Mobley’s [57] Model of Employee Turnover.	Strategy: Snowball sampling Size: Out of the 4520 nurses initially eligible, a final sample of 83 participants was used for analysis (inclusion and exclusion criteria were provided). Setting: A variety of mental health settings (regular hospitals, psychiatric hospitals, prisons, jails, outpatient mental health organizations).	Job Satisfaction Survey (JSS). Cronbach’s alpha ranged from 0.60 to 0.82 (nine aspects: pay, promotion, supervision, benefits, contingent rewards, operating procedures, coworkers, nature of work, and communication). Competing Values Managerial Skills Instrument. Cronbach’s alpha ranged from 0.76 to 0.88 (leadership behavior roles: broker role, coordinator role, director role, facilitator role, mentor role, producer role, and innovator role). Internal Validity Concern: Instrumentation: Not a problem External Validity Concern: No report on response rate.	Canonical correlation analysis	Millennial psychiatric nurses who perceived their managers to display the roles in being a facilitator (rs = 0.618), mentor (rs = 0.965), innovator (rs = 0.691), broker (rs = 0.588), producer (rs = 0.692), director (rs = 0.746), and monitor (rs = 0.749)) more frequently, tended to have higher job satisfaction in terms of:	
						Pay, contingent rewards	Benefit (Hygiene)
						Promotion	Advancement (Motivation)
						Supervision	Supervision (Hygiene)
						Coworkers	Interpersonal relationship (Hygiene)
						Nature of work	Work itself (Motivation)
						Communication	Interpersonal relationship (Hygiene)
[58] From 16–24 December 2014 Singapore	Cross-sectional study	_	Strategy: non-probability sampling “convenience sampling”. Size: 726 nurses (inclusion and exclusion criteria were provided). Setting: A tertiary psychiatric institution in Singapore.	McCloskey and Mueller Satisfaction Scale (MMSS). Cronbach alpha = 0.89. Resilience Scale. Cronbach alpha ranged from 0.72 to 0.94. Internal Validity Concern: Instrumentation: Not a problem. External Validity Concern: Response Rate: Not a problem; the study reported a response rate of 83.1%.	Linear regression	Positive association between resilience and job satisfaction (β = 0.109, t = 2.953, *p* = 0.003).	Personal/Emotional factor
[59] From 18–31 December 2017 China	Cross-sectional survey.	_	Strategy: Although not explicitly stated, the study appears to use stratified sampling, targeting specific hospitals in specific locations to ensure regional representation. Size: Among the total of 9907 nurses, a response was received from 8493 nurses (inclusion and exclusion criteria were provided). Setting: 32 tertiary psychiatric hospitals in 29 provincial capitals in China.	Short-form Minnesota Satisfaction Questionnaire (MSQ). Cronbach’s alpha was >0.7. Internal Validity Concern: Instrumentation: Not a problem. External Validity Concern: Response Rate: Not a problem; the study reported a response rate of 85.7%.	Multiple linear regression	Age was negatively associated with job satisfaction, such that the age groups of 28–32 years (B = −0.975, *p* < 0.05), 33–40 years (B = −1.973, *p* < 0.05), and 41 years and above (B = −3.191, *p* < 0.05) all had significantly lower job satisfaction scores compared to the youngest reference group (27 and below).	Personal
						Monthly income demonstrated a positive association with job satisfaction (*p* < 0.05).	Salary (Hygiene)
						Negative association between working more than 40 h per week and job satisfaction scores for nurses (B = −2.702, *p* < 0.05).	Work conditions (Hygiene)
						Social recognition showed a positive association with job satisfaction (*p* < 0.05).	Recognition (Motivation)
						Nurse–physician collaboration exhibited a positive association with job satisfaction (*p* < 0.05).	Interpersonal relationship (Hygiene)
						Experiencing violence initiated by patients, whether in the form of verbal abuse or physical assault, had a negative correlation with job satisfaction (*p* < 0.05).	Experienced violence (Psychosocial factor)
[60] N/S Egypt	Descriptive correlational design	_	Strategy: non-probability sampling “convenience sampling”. Size: A total of 600 individuals from all departments (inclusion criteria provided, exclusion criteria unclear). Setting: Al-Abbassia Mental Health Hospital (AMHH), Egypt.	-Work place Violence Scale Cronbach’s alpha coefficient = 0.90 -Job Satisfaction Scale Cronbach’s alpha coefficient = 0.87 -Work Productivity Scale Cronbach’s alpha coefficient = 0.60 Internal Validity Concern: Instrumentation: Not a problem. External Validity Concern: Response Rate: Based on the frequency distribution table, all 600 nurses returned the questionnaire. However, the authors did not explicitly state the response rate.	Correlation coefficient	There is a strong statistically significant relationship between violence and job satisfaction, with correlation coefficients of R = 0.059 and R = 0.234 at *p* = 0.000, respectively.	Workplace violence (Psychosocial factor)
						Correlation between types of aggression subscales, job satisfaction, and recurrence of violence:	Workplace violence (Psychosocial factor)
						Verbal Aggression and Job Satisfaction: R = 0.105, *p* = 0.005 (weak positive correlation, statistically significant) Verbal Aggression and Recurrence of Violence: R = 0.081, *p* = 0.005 (weak positive correlation, statistically significant) Physical Aggression Toward Things and Job Satisfaction: R = 0.145, *p* = 0.005 (weak positive correlation, statistically significant) Physical Aggression Toward Things and Recurrence of Violence: R = 0.167, *p* = 0.005 (moderate positive correlation, statistically significant) Physical Aggression Toward Self and Job Satisfaction: R = 0.168, *p* = 0.005 (moderate positive correlation, statistically significant) Physical Aggression Toward Self and Recurrence of Violence: R = 0.199, *p* = 0.005 (moderate positive correlation, statistically significant) Physical Aggression Toward Others and Job Satisfaction: R = 0.166, *p* = 0.005 (moderate positive correlation, statistically significant) Physical Aggression Toward Others and Recurrence of Violence: R = 0.206, *p* = 0.005 (stronger moderate positive correlation, statistically significant)	
[61] September to November 2021 Egypt	Descriptive correlational design	_	Strategy: Although not explicitly stated, the study seems to employ non-probability sampling, specifically convenience sampling. Size: A total of 106 out of 140 psychiatric nurses who fulfilled the study’s inclusion criteria took part in the research (inclusion criteria provided, exclusion criteria unclear). Setting: Al Abbassia mental health hospital in Cairo.	Workplace Violence in Healthcare Questionnaire (WPVHC) Cronbach’s alpha coefficient = 0.85 Perceived Stress Scale (PSS) Cronbach’s alpha coefficient = 0.94 Satisfaction of Employee in Healthcare Scale (SEHC) Cronbach’s alpha coefficient = 0.85 Internal Validity Concern: Instrumentation: Not a problem. External Validity Concern: Response Rate: Based on the frequency distribution table, it appears that all 106 nurses returned the questionnaire. However, the authors did not explicitly state the response rate.	Multiple linear regression	Statistically significant positive correlations were identified among the overall scores of workplace violence, perceived stress, and job satisfaction among the psychiatric nurses in the study (*p* < 0.001). This indicates that as exposure to workplace violence increases, both perceived stress and job satisfaction are likely to rise within this group.	Workplace violence (Psychosocial factor)

Abbreviations: N/S: not stated; MSQ: Minnesota job satisfaction questionnaire; SD: standard deviation; t: t-value; *p*: *p*-value; r: correlation coefficient; χ^2^: chi-squared statistic; KUHJSS: Kuopio university hospital job satisfaction Scale; MBI: Maslach burnout inventory; B: unstandardized coefficient; Beta: standardized coefficient; MBI-GS: Maslach burnout inventory—general survey; OR: odds ratio; JSS: job satisfaction survey; CBI: Copenhagen burnout inventory; rs: spearman rank correlation coefficient; MMSS: McCloskey and Mueller satisfaction scale; SW: Support-in-Workplace scale; WPVHC: workplace violence in healthcare questionnaire; SEHC: satisfaction of employee in healthcare scale. Factor Category Definitions: Personal: characteristics such as age, gender, education, income, and cultural background that shape an individual’s experiences and perspectives; Emotional Factors: aspects related to an individual’s feelings and psychological state, including stress, anxiety, resilience, and overall mood, which can significantly influence behavior and job satisfaction; Intrinsic Factors: internal motivators that drive an individual’s behavior and satisfaction, such as a sense of achievement, autonomy, and the enjoyment of the work itself; Extrinsic Factors: external influences that affect an individual’s motivation and job satisfaction, such as salary, work conditions, and interpersonal relationships; Psychosocial Factors: elements that involve both psychological and social aspects, including stress, workplace violence, and social support.

**Table 3:** JBI checklist for cross-sectional studies.

Study	Were the Criteria for Inclusion in the Sample Clearly Defined?	Were the Study Subjects and the Setting Described in Detail?	Was the Exposure Measured in a Valid and Reliable Way?	Were Objective, Standard Criteria Used for Measurement of the Condition?	Were Confounding Factors Identified?	Were Strategies to Deal with Confounding Factors Stated?	Were the Outcomes Measured in a Valid and Reliable Way?	Was Appropriate Statistical Analysis Used?
[43]	+	+	+	+	–	–	+	+
[44]	Exclusion (?)	+	+	+	–	–	+	+
[45]	–	–	+	+	–	–	+	+
[47]	Exclusion (?)	+	+	+	–	–	+	+
[22]	Exclusion (?)	+	+	+	–	–	+	+
[48]	+	+	+	+	+	+	+	+
[49]	+	+	+	+	–	–	+	+
[50]	Exclusion (?)	+	?	+	–	–	?	+
[51]	+	+	+	+	+	+	?	+
[53]	+	+	+	+	–	–	+	+
[54]	+	+	?	+	–	–	?	+
[55]	+	+	+	+	–	–	+	+
[58]	+	+	+	+	–	–	+	+
[59]	+	+	+	+	?	?	+	+
[60]	Exclusion (?)	+	+	+	–	–	+	–
[61]	Exclusion (?)	+	+	+	–	–	+	–

+: yes, –: no, ?: unclear (based on the JBI checklist criteria).

## Data Availability

Data are contained within the article.
